# Effects of Anti-VEGF on Predicted Antibody Biodistribution: Roles of Vascular Volume, Interstitial Volume, and Blood Flow

**DOI:** 10.1371/journal.pone.0017874

**Published:** 2011-03-15

**Authors:** C. Andrew Boswell, Gregory Z. Ferl, Eduardo E. Mundo, Daniela Bumbaca, Michelle G. Schweiger, Frank-Peter Theil, Paul J. Fielder, Leslie A. Khawli

**Affiliations:** 1 Department of Pharmacokinetic and Pharmacodynamic Sciences, Genentech Inc., South San Francisco, California, United States of America; 2 Department of Investigative Safety Assessment, Genentech Inc., South San Francisco, California, United States of America; University of Texas, M.D. Anderson Cancer Center, United States of America

## Abstract

**Background:**

The identification of clinically meaningful and predictive models of disposition kinetics for cancer therapeutics is an ongoing pursuit in drug development. In particular, the growing interest in preclinical evaluation of anti-angiogenic agents alone or in combination with other drugs requires a complete understanding of the associated physiological consequences.

**Methodology/Principal Findings:**

Technescan™ PYP™, a clinically utilized radiopharmaceutical, was used to measure tissue vascular volumes in beige nude mice that were naïve or administered a single intravenous bolus dose of a murine anti-vascular endothelial growth factor (anti-VEGF) antibody (10 mg/kg) 24 h prior to assay. Anti-VEGF had no significant effect (*p*>0.05) on the fractional vascular volumes of any tissues studied; these findings were further supported by single photon emission computed tomographic imaging. In addition, apart from a borderline significant increase (*p* = 0.048) in mean hepatic blood flow, no significant anti-VEGF-induced differences were observed (*p*>0.05) in two additional physiological parameters, interstitial fluid volume and the organ blood flow rate, measured using indium-111-pentetate and rubidium-86 chloride, respectively. Areas under the concentration-time curves generated by a physiologically-based pharmacokinetic model changed substantially (>25%) in several tissues when model parameters describing compartmental volumes and blood flow rates were switched from literature to our experimentally derived values. However, negligible changes in predicted tissue exposure were observed when comparing simulations based on parameters measured in naïve versus anti-VEGF-administered mice.

**Conclusions/Significance:**

These observations may foster an enhanced understanding of anti-VEGF effects in murine tissues and, in particular, may be useful in modeling antibody uptake alone or in combination with anti-VEGF.

## Introduction

The absence of many physiological processes *in vitro* and interspecies differences *in vivo* can confound direct comparisons of *in vitro*, preclinical, and clinical data [1], [2]. A vast array of physiological data for humans and laboratory species is available in the literature [3], [4], [5], [6]; however, it should be utilized with an understanding of its limitations. Significant physiological variability across species, age, breed, disease status, drug treatment, and time of day [7] motivates direct measurement of relevant physiological properties or processes whenever possible [8].

The measurable effects of anti-vascular endothelial growth factor (anti-VEGF) therapy on tumors in preclinical and clinical settings include reductions in vascular density, vascular volume, vessel permeability, and/or blood flow [9]; however, changes have also been reported for small molecule VEGF inhibitors in non-malignant tissues [10]. For instance, inhibition of VEGF in mice using non-antibody-based anti-angiogenic agents induced distinct physiological changes, including reduced cardiac output, changes in blood glucose regulation, reduction of endothelial cell fenestrations, and significant capillary regression in several tissues [10]. Several VEGF inhibitors have yielded measureable, although generally manageable, adverse effects in the clinical oncology setting [11], thus encouraging further studies into the underlying mechanisms behind the observed biological changes.

**Figure 1:**
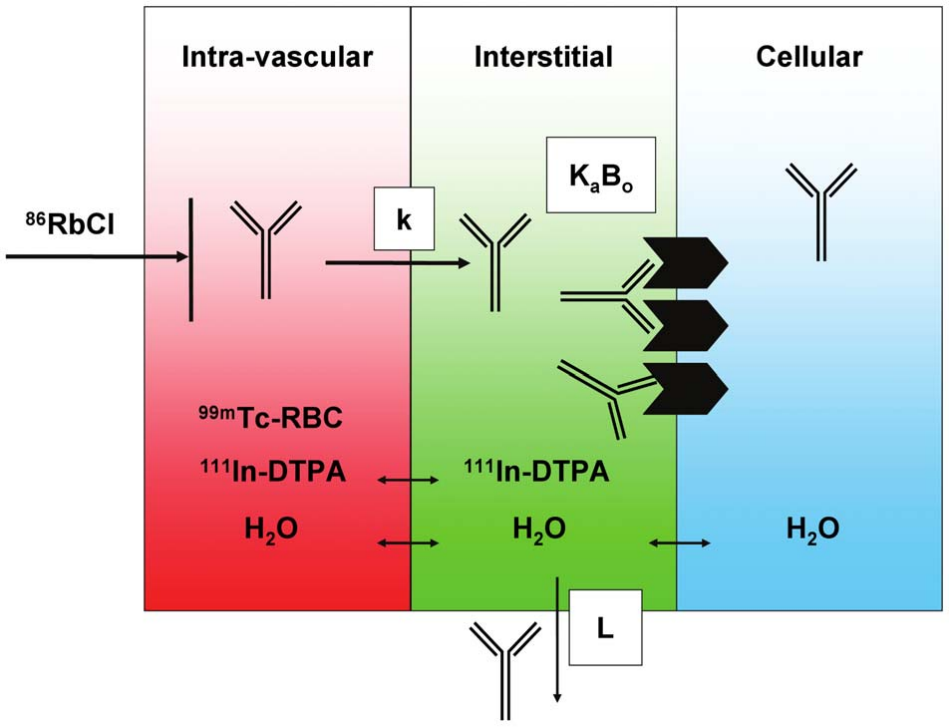
Conceptual illustration of techniques used to measure physiological parameters relevant to antibody uptake in tissues. The tissue is divided into vascular, interstitial, and cellular compartments (depicted in red, green, and blue, respectively). The blood space (*V_v_*) may be measured using ^99m^Tc-labeled red blood cells (RBC), while the extracellular (i.e. *V_v_*+*V_i_*) space is measured by infusion of ^111^In-DTPA. The rate of blood flow (*Q*) to the tissue may be measured as the proportion of a bolus dose of ^86^RbCl that enters the tissue in a brief time interval. The antibody's receptor, if present, may be expressed on the cell surface, exposed to the interstitial fluid. An antibody in circulation may extravasate from blood into interstitial space at a rate (k), where it may encounter a number (B_o_) of receptors for which it has binding affinity (K_a_). The antibody may also return to circulation via lymphatic flow (L).

Preclinical studies evaluating the effects of anti-angiogenic antibodies on all aspects of tissue physiology may have important implications, especially given the increased clinical interest in antibodies against angiogenic targets [9]. The present study investigates the impact of a cross-species anti-VEGF antibody [12], B20-4.1, on the vascular volumes (*V_v_*), interstitial fluid volumes (*V_i_*), and regional rates of blood flow (*Q*) for selected tissues in nude mice (**Figure 1**).

**Figure 2:**
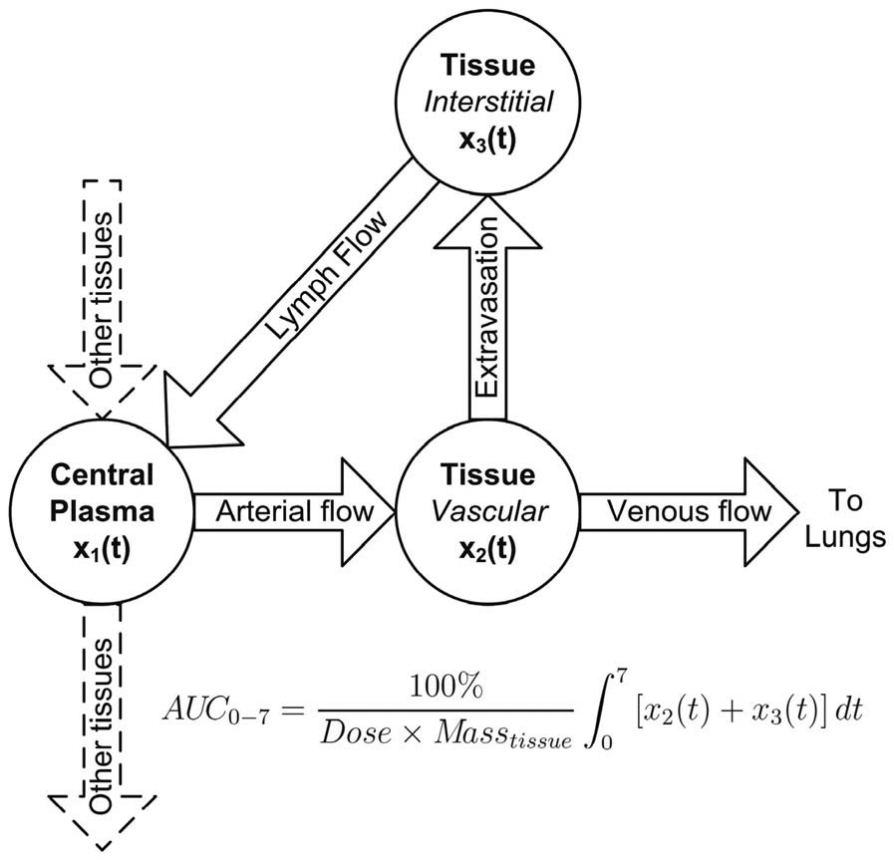
Diagram of physiologically-based pharmacokinetic (PBPK) model to predict antibody uptake in tissues. Shown is a typical tissue sub-model component of the PBPK model [13] used to assess the influence of parameter variability among literature and measured *V_v_*, *V_i_* and *Q* values on tissue uptake of an IgG (expressed as AUC_0–7_). Antibody enters tissue from the central plasma compartment via arterial blood flow where it continues to the lungs via venous blood flow or returns directly to the central plasma compartment through the lymphatic system subsequent to extravasation into interstitial space. The AUC_0–7_ values listed in **Table 4** are the sum of AUCs of absolute antibody amount vs. time in the two tissue compartments (x_2_ and x_3_) multiplied by 100% and divided by the product of the total injected dose and mass of tissue, yielding AUC in units of %ID/g × time. Note that the muscle sub-model includes extra compartments, included in the AUC_0–7_ calculation, that describe FcRn mediated recycling and degradation of antibody.

Physiologically-based pharmacokinetic (PBPK) modeling can aid in understanding mechanisms of tissue uptake and can predict, by means of inter-species scaling, tissue concentrations of therapeutic antibodies in humans based on preclinical PK data of molecules currently in development [13]. The success of PBPK modeling, however, is dependent on parameter values that accurately reflect *in vivo* tissue physiological conditions. Importantly, a sensitivity analysis of a previously reported PBPK model implicated *V_v_* and *V_i_* as two of the most influential parameters on antibody concentration in tissues, particularly at early time points [5]. In addition, preclinical and clinical magnetic resonance imaging studies have demonstrated changes in parameters describing *V_v_* and *Q* and/or vessel permeability in tumors following anti-VEGF treatment [9]. In this context, tissue uptake of a generic IgG was predicted by physiologically-based pharmacokinetic (PBPK) modeling (**Figure 2**) using *V_v_*, *V_i_* and *Q* values obtained from the literature, measured in naïve mice, or measured in mice receiving anti-VEGF; predicted uptake values were compared to experimental uptake data for a model antibody (trastuzumab) in nude mice.

## Results

### Vascular volume

**Figure 3:**
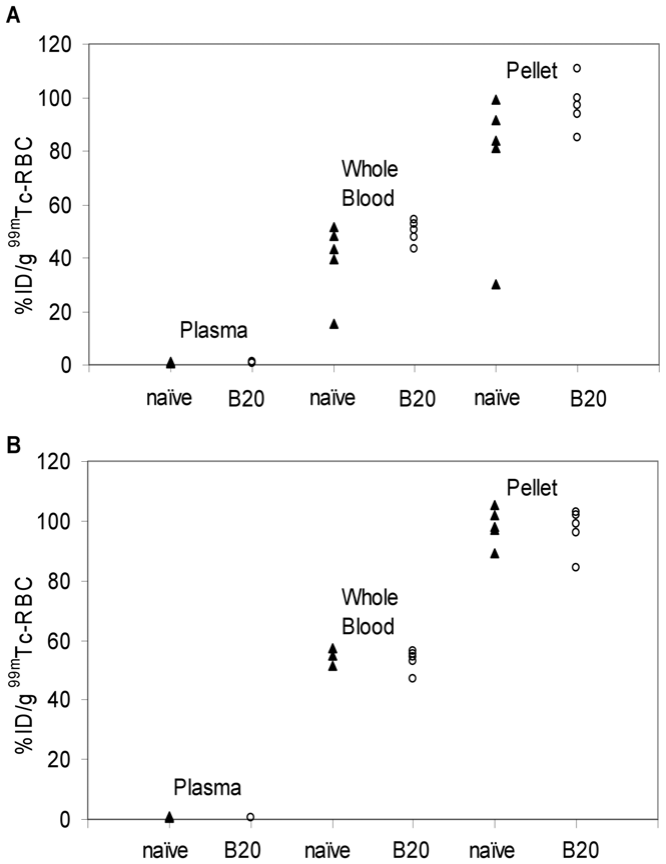
Measurement of technetium-99m incorporation in fractionated red blood cells. (A) Technetium-99m radioactivity, expressed as percentage of injected ^99m^Tc dose per gram (%ID/g), in fractionated blood for mice (n = 5) whose red blood cells were labeled by the direct method. Mice were either naïve or administered a single intravenous bolus dose (10 mg/kg) of the cross-species anti-VEGF antibody, B20-4.1, 24 h prior to assay. (B) Technetium-99m radioactivity, expressed as %ID/g, in fractionated blood from mice (n = 5) whose red blood cells were labeled by the indirect method. All donor mice were naïve; recipient mice were naïve or received a single intravenous bolus dose (10 mg/kg) of the cross-species anti-VEGF antibody, B20-4.1, 24 h prior to assay.

Successful RBC labeling with ^99m^Tc was evident due to observed association of the vast majority of radioactivity with the RBC pellet fraction for both naïve and B20-4.1-administered mice (**Figure 3**). For the direct RBC labeling method, the mean %ID/g values for the naïve and B20-4.1-administered mice, respectively, were 0.79±0.14 vs. 0.75±0.11 in plasma, 39.6±14.2 vs. 49.7±4.4 in whole blood, and 77.1±27.3 vs. 97.3±9.3 in the RBC pellet. To ensure that the anti-angiogenic effects did not interfere with the measurement, a refined indirect method for measuring *V_v_* [2] allowed red blood cell (RBC) labeling to be performed in a separate cohort of naïve (i.e. receiving no anti-VEGF) mice. For the indirect method, the mean %ID/g values for the naïve and B20-4.1-administered mice, respectively, were 0.63±0.19 vs. 0.57±0.13 in plasma, 55.1±2.5 vs. 53.3±3.6 in whole blood, and 98.3±6.2 vs. 96.8±7.5 in the RBC pellet.

Mean values with standard deviations were calculated for direct and indirect *V_v_* data from both dose groups and compared to literature values (**Table 1**). Using the direct method, no differences in *V_v_* for brain and muscle were observed when comparing naïve and B20-4.1-administered mice. Differences, expressed as [(*V_v,B20-4.1_*−*V_v,naïve_*)/*V_v, naïve_*]×100%, were noted in spleen (5% increase), liver (11% decrease), kidney (27% decrease), intestine (22% decrease), heart (9% decrease), lung (11% decrease), and fat (7% increase). None of these differences were statistically significant by unpaired *t*-test (*p*>0.05). Using the indirect method, no differences in *V_v_* were observed between naïve and B20-4.1-administered mice for brain, muscle, and fat. Percent differences for remaining tissues, expressed as [(*V_v,B20-4.1_*−*V_v,naïve_*)/*V_v, naïve_*]×100%, were as follows: spleen (9% increase), liver (1% decrease), kidney (7% decrease), intestine (30% increase), heart (27% increase), and lung (21% decrease). None of these differences was statistically significant by unpaired *t*-test (*p*>0.05).

**Table 1:**
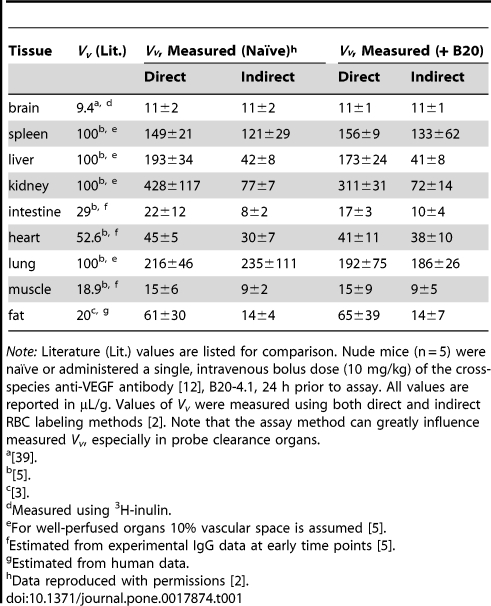
Measured vascular volumes (*V_v_*) in naïve and anti-VEGF-administered mice.

*Note:* Literature (Lit.) values are listed for comparison. Nude mice (n = 5) were naïve or administered a single, intravenous bolus dose (10 mg/kg) of the cross-species anti-VEGF antibody [12], B20-4.1, 24 h prior to assay. All values are reported in µL/g. Values of *V_v_* were measured using both direct and indirect RBC labeling methods [2]. Note that the assay method can greatly influence measured *V_v_*, especially in probe clearance organs.

a [39].

b [5].

c [3].

d Measured using ^3^H-inulin.

e For well-perfused organs 10% vascular space is assumed [5].

f Estimated from experimental IgG data at early time points [5].

g Estimated from human data.

h Data reproduced with permissions [2].

Excellent agreement between indirectly measured (in naïve mice) and literature *V_v_* values, respectively, was observed for brain (11±2 vs. 9.4 µL/g) and spleen (121±29 vs. 100 µL/g) (**Table 1**). In contrast, the direct method yielded *V_v_* values more closely matching the corresponding literature values for intestine (22±12 vs. 29 µL/g) and muscle (15±6 vs. 18.9 µL/g).

### SPECT-CT imaging

**Figure 4:**
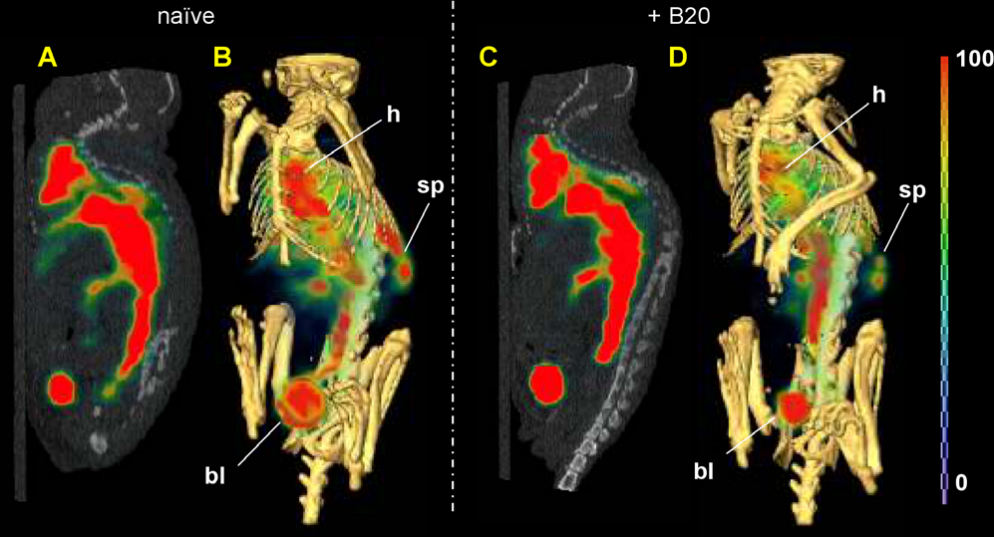
Noninvasive SPECT-CT imaging of blood pool in naïve and anti-VEGF-administered mice. Representative SPECT-CT blood pool images (n = 1) obtained at 98–138 min post injection in mice that were either naïve (A–B) or administered (C–D) a single intravenous bolus dose (10 mg/kg) of the cross-species anti-VEGF antibody, B20-4.1, approximately 24 h prior to image acquisition. Red blood cell labeling was performed by the indirect method. The false-colored SPECT images in arbitrary uptake units are fused onto the X-ray CT images. Both a sagittal planar image along the spine (A, C) and a corresponding three-dimensional volume rendered image (B, D) are shown for each reconstructed SPECT-CT fusion dataset. Mostly blood pool and bladder uptake are evident in the sagittal slices, while the spleen can also be clearly delineated in the right-hand side of the 3D images, just below the ribcage. The locations of visible uptake in heart (h), spleen (sp), and bladder (bl) are indicated in the volume rendering images.

The whole-body distributions of ^99m^Tc-labeled RBCs for the two dose groups were visually assessed by single photon emission computed tomography/X ray computed tomography (SPECT-CT) imaging. Both the sagittal planar images (left) and the three-dimensional volume rendered images (right) revealed similar blood distributions for both naïve and B20-4.1-administered mice (**Figure 4**). Slight splenic uptake was evident in the SPECT-CT volume rendered images of mice in both dose groups. It should be noted that the magnitude of bladder uptake may be affected by differences in the time between injection and the start of SPECT data acquisition (98 min for naïve, 138 min for B20-4.1-administered mouse); in contrast, the mice that were used to generate the data in **Figure 3** were promptly sacrificed at 1 h post-injection of ^99m^Tc.

### Interstitial volume

From the calculated interstitial fluid volume data in naïve and B20-4.1-administered mice, mean *V_i_* values were obtained and compared to literature values (**Table 2**). It should be noted that because the calculation of *V_i_* requires knowledge of *V_v_*, its accuracy is dependent on both the RBC labeling and indium-111 diethylenetriamine-*N,N,N′,N′,N″*-pentaacetic acid (^111^In-DTPA; i.e., ^111^In-pentetate) infusion studies. Mean *V_i_* values for B20-4.1-administered relative to naïve mice, expressed as [(*V_i,B20-4.1_*−*V_i,naïve_*)/*V_i, naïve_*]×100%, were as follows: brain (80% increase), spleen (8% decrease), liver (20% increase), intestine (25% increase), heart (5% increase), lungs (50% increase), muscle (32% increase), and fat (11% decrease). With the exception of brain, none of these differences is statistically significant by unpaired *t*-test (*p*>0.05). For brain, the difference exhibits a borderline statistical significance by unpaired *t*-test (*p* = 0.05).

**Table 2:** Measured interstitial volumes (*V_i_*) in naïve and anti-VEGF-administered mice.

Tissue	*V_i_* (Literature)	*V_i_*, Measured (Naïve)	*V_i_*, Measured (+ B20)
brain	170–190^a^^, ^ d	20±5^e^	36±15^e^
spleen	200^b^	25±10	23±32
liver	200^b^^, ^ g	90±15	108±23
kidney	339^b^^, ^ g	2202±462^f^	2588±506^f^
intestine	174^b^^, ^ h	121±62	151±96
heart	143^b^^, ^ h	158±67	166±41
lung	300^b^^, ^ g	173±71	259±111
muscle	130^b^^, ^ i	114±119	150±47
fat	490^c^^, ^ i	346±259	308±121

*Note:* Literature values are listed for comparison. Nude mice (n = 5) were naive or administered a single, intravenous bolus dose (10 mg/kg) of the cross-species anti-VEGF antibody [12], B20-4.1, 24 h prior to assay. All values are reported in µL/g. Note that the assay method can greatly influence measured *V_i_* values, especially in probe clearance organs.

a [14].

b [5].

c [23].

d Tetramethylammonium (TMA^+^) method; corrected for a reported 9.4 µL/g blood volume [14].

e Reflects inability of ^111^In-DTPA to cross the blood-brain barrier.

f Non-physiologically relevant measurement due to renal clearance of ^111^In-DTPA.

g Extrapolated from rat; sodium (extracellular marker) content determined in a flame photometer [22].

h Estimated on the basis of values for similar tissues [5].

i Extrapolated from rat; ^51^Cr-EDTA method [5], [23].

Agreement between measured (in naïve mice) and literature *V_i_* values, respectively, was observed for intestine (121±62 vs. 174 µL/g), heart (158±67 vs. 143 µL/g), muscle (114±19 vs. 130 µL/g), and fat (346±259 vs. 490 µL/g) (**Table 2**). The *V_i_* value for brain in mice (20±5 µL/g) does not agree with the literature value in mice (170–190 µL/g) [14] due to an inability of the radiometal-chelate complex to cross the blood-brain barrier (**Table 2**). Similarly, the *V_i_* value for kidneys in naïve mice (2202±462 µL/g) is also physiologically irrelevant due to renal clearance of the extracellular probe, ^111^In-DTPA (**Table 2**).

### Organ blood flow rates

From the calculated blood flow data in naïve and B20-4.1-administered mice, mean *Q* values (µL/g/min) were obtained and compared to literature values (**Table 3**). Percent differences in mean *Q* values, expressed as [(*Q_,B20-4.1_*−*Q_,naïve_*)/*Q_, naïve_*]×100%, were as follows: liver (27% increase), kidneys (19% increase), heart (17% increase), muscle (6% increase), fat (18% increase), spleen (9% decrease), intestine (9% decrease), and unchanged in lungs. With the exception of liver, none of these differences is statistically significant by unpaired *t*-test (*p*>0.05). For liver, by conventional criteria, the difference is considered to be borderline statistically significant by unpaired *t*-test (*p* = 0.048).

**Table 3:** Volume velocities of regional blood flow (*Q*) in naïve and anti-VEGF-administered mice.

Tissue	*Q*, Literature	*Q*, Measured (Naïve)	*Q*, Measured (+ B20)
brain	850^a^^, ^ e	12±1^d^	12±1^d^
spleen	909^b^^, ^ f	169±105	153±71
liver	2103^b^^, ^ f	158±20*	200±35*
kidney	4881^b^^, ^ g	1172±419	1390±537
intestine	474^b^^, ^ f	380±136	346±105
heart	3828^b^^, ^ f	877±63	1026±236
lung	350^a^^, ^ h	538±150	537±262
muscle	184^b^^, ^ i	186±47	198±59
fat	190^c^^, ^ j	120±37	142±34

*Note:* Literature values are listed for comparison. Nude mice (n = 5) were naïve or administered a single, intravenous bolus dose (10 mg/kg) of the cross-species anti-VEGF antibody [12], B20-4.1, 24 h prior to assay. All values are reported in µL/g/min. Note that the assay method can greatly influence measured values of *Q*, especially in probe clearance organs.

* *p*<0.05 by unpaired *t*-test.

a [3].

b [5].

c [40].

d Reflects inability of probe to cross blood-brain barrier.

e ^85^Sr-labeled microspheres [15].

f Origin of measurement not specified in literature.

g Extrapolated from inulin clearance rates in rat kidney [41].

h Bronchial flow.

i Measured experimentally by Rb uptake method.

j Rat data; method not specified [40].

Agreement between measured (in naïve mice) and literature *Q* values, respectively, was observed for intestine (380±136 vs. 474 µL/g/min) and muscle (186±47 vs. 184 µL/g/min) (**Table 3**) [5]. The *Q* value for brain in naïve mice (12±1 µL/g/min) does not agree with the literature value (850 µL/g/min) [3], [15] due to an inability of the radiometal cation, ^86^Rb^+^, to cross the blood-brain barrier.

### PBPK simulations

Listed in **Table 4** are the simulated AUC_0–7_ values for blood and various tissues using a PBPK model parameterized with both literature and measured values of *V_v_*, *V_i_*, and *Q*, with the exception of kidney, for which literature values of *V_i_*, were always used. The substitution of measured physiological parameter values in naïve mice resulted in changes in AUC_0–7_ values, expressed as [(AUC_0–7, *measured*_−AUC_0–7, *literature*_)/AUC_0–7, *literature*_]×100%, as follows: 35% increase in lungs, 15% increase in spleen, 13% increase in blood and bone, 48% decrease in liver, 46% decrease in intestine, 28% decrease in muscle, 5% decrease in kidneys, and 1% increase in heart. Note that no experimental bone measurements were made in this study. Literature values were used for this tissue in all cases, and changes in bone reflect altered plasma PK profiles.

**Table 4:** Sensitivity of AUC_0–7_ for an antibody in mice to changes in *V_v_*, *V*_i_ and *Q*.

Tissue	Model-Predicted AUC_0–7_ Values			Experimental^a^
	Lit.	Meas. (naïve)	Meas. (+ B20)	(naïve)
blood	82.5	93.0	93.1	122.2
liver	18.1	9.37	9.64	20.7
kidneys	18.7	17.8	17.1	21.8
spleen	15.4	17.7	19.4	28.1
lungs	36.7	49.5	50.2	33.1
heart	17.4	17.6	19.3	24.3
muscle	11.4	8.23	8.22	7.90
intestine	7.03	3.77	4.71	na
bone	7.58	8.55	8.56	na

*Note:* Comparison of model-predicted [13] and experimentally measured AUCs [%ID/g day], up to 7 days post-injection, calculated for an intact antibody in mice. All blood flow rates (*Q*), vascular (*V_v_*) and interstitial (*V_i_*) volumes were simultaneously changed from literature to measured values (see **Tables 1** **,** **2** **,** **3**) for these simulations, with the exception of *V_i_* for kidneys, for which literature values were always used due to the effects of probe clearance. Measurement of *V_v_* was accomplished using a previously reported indirect RBC labeling method [2].

a Data for ^125^I-trastuzumab; reproduced with permissions [2].

Model-predicted AUC values may also be compared to experimentally measured values obtained from tissue distribution data (**Table 4**). The use of experimentally measured *V_v_*, *V_i_*, and *Q* values in naïve mice produced simulations that more closely matched measured AUC_0–7_ values in blood, spleen, and muscle, but not in liver, kidneys, and lungs when compared to simulations based on literature values. Specifically, the use of literature and measured parameter values, respectively, gave differences, expressed as [(AUC_0–7, *model-predicted*_−AUC_0–7, *experimental*_)/AUC_0–7, *experimental*_]×100%, as follows: 44% higher vs. 4% higher in muscle, 11% higher vs. 50% higher in lungs, 45% lower vs. 37% lower in spleen, 32% lower vs. 24% lower in blood, 28% lower vs. 28% lower in heart, 13% lower vs. 55% lower in liver, and 14% lower vs. 18% lower in kidneys.

Model-predicted AUC values obtained using experimentally measured values in naïve and anti-VEGF-administered mice were very similar in blood and most tissues (**Table 4**). The use of measured parameter values in naïve and B20-4.1-administered mice, respectively, gave the following differences, expressed as [(AUC_0–7, *naïve*_−AUC_0–7, *B20-4.1*_)/AUC_0–7, *naïve*_]×100%: 25% higher in intestine, 10% higher in heart and spleen, 3% higher in liver, 1% higher in lungs, 0.1% higher in blood and bone, 4% lower in kidneys, and 0.1% lower in muscle.

## Discussion

No significant change in three measured physiological parameters was observed for most healthy murine tissues, despite the fact that the chosen dosage of B20-4.1 was based on previously reported xenograft growth inhibition activity [16] that should result in a minimum trough concentration at steady state of ∼30 µg/mL, similar to that achieved in >90% of bevacizumab patients [17]. Furthermore, selection of the 24 h time point was guided by reported statistically significant reductions in vascular density of human xenografts in mice at 24 h following anti-VEGF administration [9]. The lack of observed changes in physiology reported herein may reflect lower murine VEGF levels in healthy tissues relative to tumors in mice. Indeed, an analogous scenario has been exploited in clinical imaging of VEGF-expressing tumors with radiolabeled bevacizumab, where metastatic lesions were clearly delineated [18]. However, these findings do not preclude possible changes in additional physiological parameters such as vascular permeability to immunoglobulins; additional studies in this context are ongoing.

The blood volume measurement data indicated that the %ID/g values for whole blood were roughly half of those for the RBC pellet by either the direct or indirect labeling method, consistent with an average hematocrit value of 45% in mice [4]. Although the differences in RBC labeling efficiency between naïve and B20-4.1-administered mice were not statistically significant, the mean values in blood and RBC pellet were higher for the B20-4.1-administered mice, possibly due to anti-VEGF-mediated decrease in nitric oxide (NO) synthesis and concomitant vasoconstriction that would prolong the exposure of RBCs to Sn^2+^ and/or ^99m^Tc pertechnetate. These observations led to the development and utilization of the indirect method to ensure that such effects were not confounding the *V_v_* results [2].

The overall similarity in distributions of radioactivity between the two images (**Figure 4**) was consistent with the lack of significant anti-VEGF effect on measured *V_v_* values (**Table 1**). Slight splenic uptake of ^99m^Tc observed in SPECT-CT images was observed, as expected, due the spleen's physiological role in sequestration of RBCs [19], [20], [21]. Furthermore, anti-VEGF administration had no statistically significant effect on measured *V_i_* values (**Table 2**). Because calculation of *V_i_* requires knowledge of both vascular (^99m^Tc-RBC) and extracellular (^111^In-DTPA) spaces, statistical analysis of raw %ID/g ^111^In-DTPA data was performed separately and showed no significant differences between dose groups (data not shown).

Comparing the PBPK modeling results using literature and measured parameter values demonstrates the importance of obtaining accurate values for physiological parameters. While the change in blood AUC is modest (13%) (**Table 4**), the impact of using measured physiological values is potentially high when simulating concentration-time curves within certain organs, with changes in liver and intestine AUCs of approximately 50%. Variability in AUC_0–7_ for liver is of particular significance when modeling antibody biodistribution because of its role as a clearance organ.

Although closer agreement between model predicted and experimental AUC_0–7_ values was obtained using literature *V_v_*, *V_i_*, and *Q* values in liver, kidneys, and lungs, the use of experimentally determined parameter values gave superior results in blood, muscle, and spleen (**Table 4**). Both methods demonstrated roughly comparable performance in predicting AUC_0–7_ values in heart. The excellent agreement between model predicted and experimental values in muscle is of particular significance given the inclusion of an FcRn submodel in the muscle sub-compartment of the PBPK model [13]. This nonlinear two-compartment submodel accounts for linear transfer of antibody from organ vascular space to endosomes via nonspecific bulk fluid uptake by endothelial cells, recycling of FcRn-bound antibody back into plasma, and degradation of non-FcRn-bound antibody.

Difficulty in harvesting lungs without pooling of excess blood during sacrifice may have influenced the calculated parameter values for this tissue. In addition, clearance of physiological probes may limit the validity of measurements. This effect was avoided for the vascular volume measurement by use of an indirect RBC labeling protocol to avoid contamination by non-RBC-associated ^99m^Tc; however, ^111^In-DTPA and ^86^Rb are also subject to renal clearance and, in the case of ^111^In-DTPA, possible transchelation of radiometal into metalloproteins may encourage hepatic accumulation. Due to the aforementioned complications, utilization of nominal values for lungs, liver, and kidney may be superior to the use of measured values.

The origin of values reported in the literature (**Tables 1** **,** **2** **,** **3**) should be carefully considered, as many are in fact assumed nominal values. For instance, a 10% vascular space is assumed for well-perfused organs (*e.g.*, kidney, lung, liver, and spleen), while, other values are estimated based on experimental uptake data of antibodies at early time points [5] (**Table 1**). In addition, multiple methods exist for measuring physiological parameters. Three distinct methods were used to obtain several *V_i_* values that were derived from a single reference in **Table 2**: analysis of sodium content (in rat tissues) by flame photometry [22], estimation on the basis of similar tissues [5], and use of the extracellular probe, ^51^Cr-EDTA in rat tissues [5], [23]. As expected, the best agreement is found when similar methods are employed. For instance, the *V_i_* values for muscle and fat agree well with literature values despite the difference in species; this may be explained by similar chemical properties of the radiometal-polyaminopolycarboxylate complexes (i.e. ^111^In-DTPA and ^51^Cr-EDTA) used as extracellular markers. Furthermore, the impressively good agreement between the experimental (naïve) and literature *Q* values for muscle may be explained by the fact that the identical method of rubidium uptake was used to derive both values (**Table 3**). Interestingly, the *Q* value for kidney reported in the same reference [5] was extrapolated from inulin renal clearance rates, while the methods used to derive many other values could not be found in the original literature.

Of particular importance was the use of the indirect RBC labeling technique in the determination of *V_v_*. Application of the traditional method of direct *in vivo* RBC labeling resulted in the calculation of negative interstitial volume values for many tissues, especially clearance organs (data not shown). This may be explained by interference due to non-RBC-associated ^99m^Tc, leading to an incorrect assessment of vascular volume values. The indirect method averts such difficulties through transfusion of whole blood containing *in vivo*-purified ^99m^Tc-labeled RBC from donor mice into study mice.

Many clinically utilized drugs, including radiopharmaceuticals for noninvasive imaging of physiological response to drug therapy [24], may also be useful as probes in invasive preclinical studies. For instance, convenient kit preparations for radiolabeling of red blood cells can allow not only clinical blood pool imaging [19], [20], [21], [25] but also preclinical determination of vascular volume in tumors and other tissues [7], [26].

Limitations exist in measuring physiological quantities, especially in regards to organs involved in renal and hepatobiliary clearance. Use of tabular physiological parameter data from a single, well-referenced source is appealing due to convenience and peer acceptance; in this context, an effort was made to select literature values (**Tables 1** **,** **2** **,** **3**) from heavily cited sources that are commonly used by PBPK modelers. However, for any single physiological parameter, significant variability exists among values reported by various sources; this discrepancy is often caused by differences in experimental methodology. For many modeling and simulation applications, rough estimates of physiological parameters may suffice; therefore, the use of such data is justified. Nevertheless, those who utilize such information should be aware of the experimental methods and/or estimations used to derive measured physiological parameter values so that the limitations, with respect to accuracy of PBPK model predictions, can be known.

Nominal or *in vitro* physiological parameters are often necessary in the use of PBPK models, which can lead to better understanding and predictability of drug distribution into various tissues [3], [6], [13], [27]. PBPK models have been developed to predict *in vivo* PK solely based upon *in vitro* and *in silico* absorption, distribution, metabolism, and excretion (ADME) data together with established physiological information that describes the mammalian body [13]. Even relatively simple models can significantly improve interpretation of uptake data by allocating drug concentrations into distinct physiological compartments, such as central plasma pool and peripheral tissue. Measurable tissue physiological parameters such as fractional interstitial and blood volumes can be used in a PBPK model [13], [28], [29], [30] to facilitate estimation of other parameters that yield additional insight into drug PK beyond what is apparent from traditional tissue distribution studies alone. For instance, correction of tissue disposition data for the fraction of drug in the vascular compartment is possible if the blood PK and tissue *V_v_* are known; this is particularly helpful for drugs having an interstitial or cellular site of action (i.e. biophase).

In conclusion, responses to a single anti-VEGF treatment were assessed by measuring three distinct physiological parameters in nude mice. Administration of anti-VEGF had no statistically significant effect on the fractional vascular volumes of any of the tissues studied, and these findings were further supported by SPECT imaging. In addition, with the exception of a marginally significant increase in hepatic blood flow, no anti-VEGF-induced differences were detected in interstitial fluid volume and organ blood flow rates. Furthermore, PBPK model-predicted AUC_0–7_ values of an IgG were in better agreement with experimental AUC_0–7_ values in blood, spleen, and muscle when using experimentally measured compartmental volume and blood flow values when compared to simulations based on literature values. These observations may have important implications in the mechanistic understanding and prediction of antibody uptake alone or in combination with anti-VEGF therapy.

## Materials and Methods

### Radiopharmaceuticals

Rubidium-86 chloride (^86^RbCl) was purchased from PerkinElmer® Life and Analytical Sciences, Inc. (Waltham, MA). Indium-111-DTPA (*i.e.* ^111^In-pentetate) and technetium-99m (^99m^Tc) pertechnetate were purchased from Covidien Radiopharmacy (South San Francisco, CA). All three were diluted with sterile normal saline prior to intravenous dosing. Technescan™ PYP™ (i.e. stannous pyrophosphate + ^99m^Tc) kits for preparation of ^99m^Tc pyrophosphate injection were purchased from Covidien Radiopharmacy (South San Francisco, CA) and used according to label instructions for the *in vivo* method of blood pool imaging with the appropriate human-to-mouse scaling.

### Animal model

The protocol, housing, and anesthesia were approved (Protocol numbers 09-2490 and 09-1245) by the Institutional Animal Care and Use Committees of Genentech Laboratory Animal Resources, in compliance with the Association for Assessment and Accreditation of Laboratory Animal Care regulations. Female beige nude X-linked immunodeficient (XID) (bg.nu.xid Harlan Laboratories) mice in a 6–8-week age range were used for all measurements. Selected mice received B20-4.1, a cross-species anti-VEGF murine antibody [12], which was intravenously administered in a 10 mg/kg bolus dose 24 h prior to the *V_v_* measurement; otherwise, consistency in handling of all mice was exercised. Selection of the B20-4.1 dose was based on previously reported xenograft growth inhibition activity at weekly doses of 10 mg/kg in immunocompromised mice [16]. In addition, a pharmacokinetic model simulation indicated that either a 5 mg/kg twice a week or 10 mg/kg weekly dosing regimen would result in a minimum trough concentration at steady state of ∼30 µg/mL, similar to that achieved in >90% of bevacizumab patients [17]. Selection of the 24 h time point was guided by reported statistically significant reductions in vascular density of human xenografts in mice at 24 h following anti-VEGF administration [9].

### Vascular volume

The intravascular spaces of murine tissues were measured using both direct and indirect methods as previously described [2]. The indirect method involved transfusion of radiolabeled blood from donor mice into study (i.e. recipient) mice, where donor mice had been subjected to ^99m^Tc labeling of RBCs *in vivo* following administration of stannous (Sn^2+^) pyrophosphate [7], [25]. Use of the clinical Technescan™ PYP™ kit is conceptually based on the original method of Sands for *in situ* (i.e. *in vivo*) RBC labeling with ^99m^Tc [26], [31]. The previous administration of stannous pyrophosphate, a component of the reconstituted Technescan kit, reduces ^99m^Tc pertechnetate intracellularly so that it may bind to the beta chain of hemoglobin [32].

### Interstitial volume

The extracellular spaces of murine tissues were measured by continuous infusion (via jugular cannulation) of the extracellular marker, ^111^In pentetate [33], [34]. A similar radiometal-polyaminopolycarboxylate complex, chromium-51- ethylenediaminetetraacetic acid (^51^Cr-EDTA), has been previously used by others in a similar context [23]. Jugular cannulation surgeries were performed on all mice 48 h prior to measurement to allow recovery while maintaining a constant saline infusion at 20 µL/h. One group of mice received a single intravenous bolus 10 mg/kg dose of B20-4.1 24 h prior to measurement. Extracellular volume measurement was achieved by administering a constant intravenous infusion of ^111^In pentetate (3700 kBq/mL) at a rate of 300 µL/h for exactly 1 h. Plasma and tissue samples were collected by retroorbital bleed and terminal organ harvest, respectively, and counted for radioactivity using a 1480 WIZARD™ Gamma Counter (Wallac, Turku, Finland) in the energy window for the 245-keV photon peak of ^111^In (t_1/2_ = 2.8 d) and with automatic background and decay correction. The interstitial volume (*V_i_*) was calculated as [(*cpm_TOTAL_* of ^111^In in 1 g tissue) − (*cpm* of ^111^In in 1 µL of blood) × (*V_v_*)]/(*cpm* of ^111^In in 1 µL of plasma).

### Volume velocity of organ blood flow

Regional blood flow rates (*Q*) in various organs and tissues were determined by sacrificing mice exactly 1 min following IV (caudal) bolus injection of a 185-kBq quantity of ^86^RbCl [7], [31], [35], [36], [37], [38]. Tissue samples were promptly collected by terminal organ harvest and counted for radioactivity using a 1480 WIZARD™ Gamma Counter in the energy window for the 1077-keV photon peak of ^86^Rb (t_1/2_ = 18.7 d) and with automatic background and decay correction. Blood flow (*Q*) was calculated as (*cpm* of ^86^Rb in 1 g tissue × CO_total_)/(*cpm* of ^86^Rb in the total injected dose), where total cardiac output (CO_total_) = 8000 µL/min for a mouse [4].

### SPECT-CT imaging

SPECT-CT imaging of mice with ^99m^Tc-labeled RBCs was performed as an adjunct to the *ex vivo* biodistribution studies as previously described [2]. All mice received a bolus intravenous dose consisting of ^99m^Tc pertechnetate in 100 µL of saline for the direct method, or a 200 µL aliquot of ^99m^Tc-labeled whole blood via the indirect method, with total injected radioactivity in the range of 10.3-14.4 MBq. Initiation of noninvasive SPECT-CT imaging was executed 98–138 min from the time of tracer injection. Image acquisition times for CT and SPECT were an average of 3 min and 12 min, respectively, with approximately 20,000 counts per projection (20 s per projection) for the latter. Calculated *V_v_* for all tissues in the imaged mice (data not shown) agreed well with the data in **Table 1**. The SPECT and CT data was transferred to a 256×256 matrix and fused using AMIRA® graphics software (TGS, San Diego), yielding simultaneous scintigraphic and anatomic information in all tomographic scans in the 3 different spatial axes.

### PBPK simulations

In order to assess the potential impact of parameter variability on physiologically-based modeling results, organ level antibody biodistribution, subsequent to a bolus dose, was simulated using a previously published PBPK model [13] (**Figure 2**). The area under the first seven days of the concentration-time curve (AUC_0–7_) for plasma and selected tissues was calculated using parameter values taken from the literature as well as with the experimentally measured values (**Tables 1** **,** **2** **,** **3**) discussed herein. Note that the literature *V_v_* and *V_i_* values in **Tables 1** **,** **2** were derived from reported fractional blood volumes (*γ*, where *γ* = *V_v_*/*V_tissue_*) and fractional interstitial fluid volumes (*φ*, where *φ* = *V_i_*/*V_tissue_*) by assuming a tissue density of 1 g/mL, consistent with assumptions made in the reference from which they were obtained [5]. Comparisons of AUC_0–7_ values were made by substituting literature values of *V_v_*, *V_i_* and *Q* for liver, kidneys, intestine, spleen and lungs with experimentally measured (AUC_0–7_) values [2]. One exception was made in that the literature value of *V_i_* for kidney was used in all cases due to renal clearance of ^111^In-DTPA.
